# Neurokinin-1 Receptor Antagonists against Hepatoblastoma

**DOI:** 10.3390/cancers11091258

**Published:** 2019-08-28

**Authors:** Miguel Muñoz, Marisa Rosso, Rafael Coveñas

**Affiliations:** 1 Hospital Infantil Virgen del Rocío, Unidad de Cuidados Intensivos Pediátricos, Av. Manuel Siurot s/n, 41013 Sevilla, Spain; 2 Institute of Neurosciences of Castilla y León (INCYL), Laboratory of Neuroanatomy of the Peptidergic Systems, University of Salamanca, 37007 Salamanca, Spain

**Keywords:** NK-1 receptor, substance P, hepatoblastoma, aprepitant, antitumor, apoptosis, angiogenesis

## Abstract

Hepatoblastoma (HB) is the most common malignant liver tumor that occurs during childhood. The prognosis of children with HB is favorable when a complete surgical resection of the tumor is possible, but for high-risk patients, the prognosis is much worse. New anti-HB strategies must be urgently developed. The undecapeptide substance P (SP) after binding to the neurokinin-1 receptor (NK-1R), regulates cancer cell proliferation, exerts an antiapoptotic effect, induces cell migration for invasion/metastasis, and triggers endothelial cell proliferation for neoangiogenesis. HB samples and cell lines overexpress NK-1R (the truncated form) and SP elicits HB cell proliferation. One of these strategies could be the use of non-peptide NK-1R antagonists. These antagonists exert, in a concentration-dependent manner, an antiproliferative action against HB cells (inhibit cell proliferation and induce the death of HB cells by apoptosis). NK-1R antagonists exerted a dual effect in HB: Decreased both tumor volume and angiogenic activity. Thus, the SP/NK-1R system is an important target in the HB treatment and NK-1R antagonists could act as specific drugs against HB cells. In this review, we update and discuss the use of NK-1R antagonists in the treatment of HB.

## 1. Introduction

Hepatoblastoma (HB) is the most frequent primary malignant liver tumor in children (generally between six months to three years). In western countries, the annual incidence is 1.2 cases per 1 million children [1]. The disease stage is the current key to the patient outcome. The prognosis of children with HB is favorable in the cases of lower risk, having a five-year event-free survival rate of 80%; however, in the group of high risk, or after relapse, survival decreases by 30–40% [2]. Unfortunately, for 25% of the patients with metastasis, overall survival remains extremely poor [3]. Therefore, it is urgent to identify new therapeutic targets and to develop new clinical strategies to improve the HB patient outcome.

**Figure 1:**
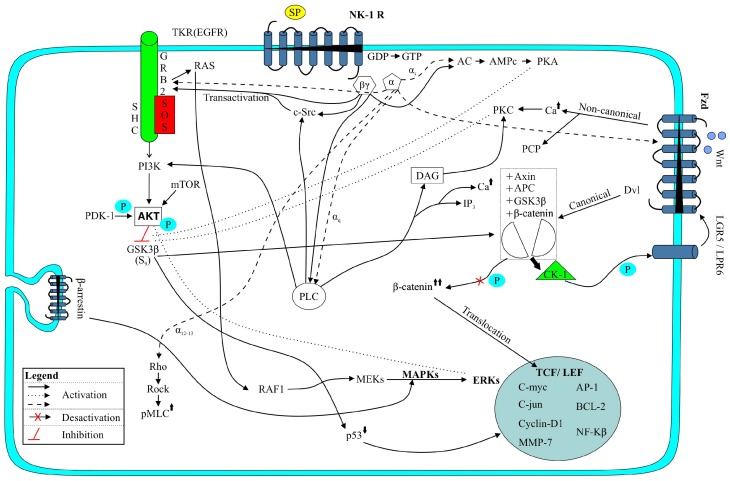
The substance P/neurokinin-1 receptor (SP/NK-1R) system regulates several cell signaling pathways involved in cancer progression. These pathways are the following: (a) Antiapoptotic signaling pathway (PI3K/Akt/mTOR); (b) cell proliferation signaling pathway (PKC, MAPKs, ERK); (c) cell migration signaling pathway (Rho-ROCK-pMLC); (d) Wnt signaling pathway (β-catenin, c-myc, cyclin D1); (e) AC-cAMP-PKA phosphorylation signaling pathway, and (f) the Warburg effect (GSK-3β). APC: Adenomatous polyposis coli; Dvl: Dishevelled; Fzd: Frizzled receptor; PCP: Planar cell polarity pathway; PDK-1: Phosphoinositide-dependent kinase-1; pMLC: Myosin light-chain kinase; TCF/LEF: Transcription factor/lymphoid enhancer-binding factor.

It is well known that some peptides and their receptors are involved in cancer. This is the case of the substance P (SP)/neurokinin-1 receptor (NK-1R) system (Figure 1), since SP, via the NK-1R, acts as a universal mitogen in human cancer cells (including HB) (Table 1) [4,5,6]. SP also exerts an antiapoptotic effect in tumor cells, induces the migration of these cells and elicits neovascularization [4]. Human tumor cells (including HB) overexpress NK-1R, this receptor being involved in the viability of these cells; this means that NK-1R can be considered a new tumor marker [4,5,6]. This is crucial, since it is also well known that NK-1R antagonists (currently, there are more than 300 compounds: Such as the drug aprepitant, L-732,138, L-733,060) exert multiple antitumor activities (antiproliferative, apoptotic effect, antimigration, antiangiogenesis) [7]. Due to the large amount of data confirming that the SP/NK-1R system is involved in cancer, here we review the involvement of this system in the HB development and we also suggest a new therapeutic strategy against HB: The use of NK-1R antagonists.

## 2. The SP/NK-1R System

The undecapeptide SP, hemokinin-1, neurokinin A and B belong to the tachykinin peptide family and, via NK-1R, NK-2R and NK-3R, many physiological actions are exerted: NK-1R shows a preferential affinity for SP/hemokinin-1, NK-2R for neurokinin A and NK-3R for neurokinin B [7]. The NK-1R protein is encoded by the *TACR1* gene (located in chromosome 2); the receptor belongs to the 1 (rhodopsin-like) G protein-coupled receptors family (also known as 7TM receptors, seven-transmembrane domain receptors or serpentine receptors) and can be coupled to several groups of G proteins: Gαi, Gαs and Gαq (Figure 1) [8,9]. The activation of a determined G protein is regulated by the conformation of the receptor as well as the type of ligand [10,11]. The G proteins differ in their signaling pathway/effectors that they activate (Figure 1) [12]. Thus, the coupling of NK-1R with the Gαi protein inhibits activity of the adenylate cyclase and decreases the level of cyclic adenosine monophosphate [13,14], whereas coupling of NK-1R with the Gαs protein activates the adenylate cyclase, the production of cyclic adenosine monophosphate, the activation of the protein kinase A and the phosphorylation of specific substrates (Figure 1) [15]. The coupling of NK-1R with the Gαq protein promotes the activation of phospholipase Cβ, an increase in the phosphatidylinositol-3 kinase, the release of diacylglycerol and an increase in the intracellular level of Ca^++^ (Figure 1) [16]. Through these pathways, the transcription of specific genes is regulated.

Seven-transmembrane-helix receptors share the same structural unit (Figure 1): An amino-terminal extracellular domain (responsible for the specificity of the receptor), a carboxy-terminal cytoplasmic domain (the carboxy-terminal conserved domain of tachykinins (Phe-X-Gly-Leu-Met-NH_2_) interacts with the receptor), and three extracellular (EL1, EL2, EL3) and intracellular (C1, C2, C3) loops flanked by seven intermembrane domains [17]. The second and third loops are involved in the binding of the SP agonists to residues 178–183 (Val-Val-Cys-Met-Ile-Glu) located in the middle of the second extracellular loop (EL2): A covalent link occurs between SP and the methyl group of a methionine residue (Met-181) [18]. The third cytoplasmic loop (C3) is responsible for the binding to protein G. The C-terminus contains serine/threonine residues, which once phosphorylated, cause desensitisation/internalization of the receptor, the latter being recycled to the plasma membrane [19]. Internalization of the NK-1R depends on the concentration of SP: Low concentration, the receptor is internalized and quickly recycled to the plasma membrane but, at high concentration, the mechanism is slower (endocytosis into endosomes) [20]. Moreover, it has been reported that the loss of certain C-terminal serine/threonine residues is important for the G protein-coupled receptor kinase interaction and β-arrestin recruitment for subsequent receptor internalization (Figure 1) [21].

Two isoforms of NK-1R have been reported: The truncated (tr-NK-1R) and the full-length (fl-NK-1R) [6,22,23]. The first contains 311 amino acids (at the C-terminus, the last 96 amino acids are lost: A premature stop codon does not allow that intron between exons 4/5 to be removed) [12] and the second one has 407 amino acids. The loss of the last 96 amino acids has been related to a loss of internalization [24]. The tr-NK-1R is able to prolong the responses after the binding of the ligands (due to the absence of a rapid desensitization) and, due to the different structure of both isoforms, it seems that they have a different functional significance, differing in cell signaling capability [25]. The tr-NK-1R promotes a rapid and sustained Ca^++^ response [24].

The presence of NK-1R has been reported in many human cancer cell lines/tissue samples and, after application of a knockdown gene-silencing method, it has been demonstrated that NK-1R plays a crucial role in the viability of tumor cells [5,26]. It seems that the degree of malignancy of a tumor correlates with the number of NK-1Rs: Greater number, higher malignancy [27] and that a poorer prognosis correlates with NK-1R overexpression [4,28]. Moreover, it is known that in malignant tissues the expression of NK-1R mRNA increased, but not in the benign tissues [27,29].

SP and the NK-1R have been observed in HB samples and the NK-1R in human HB HepT1, HepG2 and HuH6 cell lines; these three cell lines expressed mRNA for the NK-1R, and it is known that the expression of the *TAC1R* gene was increased approximately by 7.5–30 fold in HB cell lines compared to fibroblasts (Table 1 and Table 2) [6]. It is important to note that the above human HB cell lines expressed both isoforms of the NK-1R (fl-NK-1R and tr-NK-1R), but human HB HuH6, HepT1 and HepG2 cell lines expressed essentially tr-NK-1R and, in comparison to these cell lines, the fl-NK-1R expression was highest in the human embryonic kidney (HEK)-293 cells and human fibroblasts (Table 1) [6]. By contrast, the latter two cells showed a reduced number of the tr-NK-1R form [6]. It is important to note that the HEK-293 cells and fibroblasts, when studying cell survival, showed the strongest resistance to aprepitant [12]. This suggests that the action of the NK-1R antagonists is related to the differential expression of the NK-1R [12]. Moreover, it seems that the tr-NK-1R form remains up-regulated and activated continuously (as indicated above, it seems that this isoform is not internalized) and for this reason the tr-NK-1R isoform has been linked to cancer progression [25]. It has also been reported that the expression of the full-length isoform was inversely associated with proliferation, invasiveness and metastasis and that the overexpression of the tr-NK-1R isoform promotes a malignant transformation of cells, tumor progression and metastasis [30]. It is also important to note that in patients with cancer, compared to healthy subjects, the level of SP in the serum was higher [31] and that the level of the undecapeptide was > 7-fold in cultured cancer cells than in cultured normal epithelial cells [29]. Finally, in 47 children with HB, it was reported that the tr‑*TACR1* isoform was expressed ubiquitously among the different subsets of HB (Table 2). This finding is also important since the NK-1R may serve as a therapeutic target in HB patients, independent of the clinical stage/tumor biology [32]. Moreover, the tr-*TACR1* was more expressed in children with HB than in non-tumor controls, but the expression of the fl-*TACR1* showed no difference between the two groups [12]. Thus, it seems that the tr-NK-1R but not the fl-NK-1R is associated with cancer [12].

## 3. Involvement of the SP/NK-1R System in HB Progression

SP, via the NK-1R, acts as a universal mitogen in normal and tumor cells (e.g., HB, neuroblastoma, melanoma, retinoblastoma, glioma, osteosarcoma) (Table 1) [5,6,33]. In tumor cells, SP promotes c-myc mRNA and protein synthesis, c-myc being crucial for the progression through the S phase to G2/M in the cell cycle (Figure 1) [34,35]. It has been reported that c-myc is necessary to sustain a rapid tumor growth [36]. SP, via NK-1R, promotes the formation of an activated epidermal growth factor receptor (EGFR) complex, induces the transactivation of EGFR, activates the mitogen-activated protein kinase (MAPK) pathway, induces the activation of extracellular signal-regulated kinase (ERK)2, and the synthesis of DNA occurs (Figure 1) [37]. According to these data, it seems that the signaling pathways between the NK-1R and growth factors (e.g., EGFR) converge at an upstream point, favouring the pathways by which SP regulates cell proliferation [37]. It is known that c-Src interacts with EGFR inducing its phosphorylation and that the increased activity/overexpression of c-Src is related to cancer progression (Figure 1), since its interaction with EGFR/Her2 enhances mitogenic signaling pathways [38]. 

SP is synthesized/secreted by non-tumor and tumor cells; it can be released from the tumor mass into blood vessels and/or released from nerve terminals (this is important since SP regulates tumor growth and this means that an interaction between cancer cells and the nervous system occurs) [4,7]. Since several years ago, the involvement of the SP/NK-1R system in cancer (e.g., melanoma, retinoblastoma, glioma) [4,5,7] is known; however, there are few original data on the involvement of this system in HB (Table 1 and Table 2). In fact, only one review [12] and two research papers [6,32] have been currently published on the field. Thus, there are many scientific questions to be solved in the future on the involvement of the SP/NK-1R system in HB (Table 3). SP has been observed in the HB samples; the undecapeptide (at nanomolar concentration) promotes the proliferation of the HB cell lines (HepT1, HepG2, HuH6) and NK-1R antagonists (e.g., aprepitant, L-732,138, L-733,060) counteract the mitogenic action exerted by SP (Table 1 and Table 2) [6]. SP, via the NK-1R, promotes the DNA synthesis in cancer cells, activating members of the MAPK family (p38MAPK, ERK1/2) (Figure 1) [34]. Then, ERK1/2 is translocated into the nucleus, promoting cell proliferation and exerting an antiapoptotic effect [21]. 

### 3.1. PI3K/Akt/mTOR Signaling Pathway

This pathway is involved in cell proliferation and survival and it is activated via the receptor tyrosine kinase, which can be activated by cytokines or the epidermal growth factor (Figure 1) [12]. The activation of this pathway (crucial to drug resistance) promotes the activation, through phosphorylation, of many intracellular proteins. In experimental animals, it has been reported that the inhibition of mTOR affects the β-catenin/Yes-associated protein 1-induced HB development and growth [39]. The antiapoptotic effect of SP has been demonstrated in many cells (e.g., macrophages, neutrophils, thymocytes) and it is known that SP increases the phosphorylation and activity (via PI3K) of Akt (protein kinase B) (Figure 1); this activation suppresses apoptosis [11,21,40,41]. In macrophages, SP activates the PI3K/Akt/mTOR/S6kinase pathway [42], the activation of the PI3K/Akt/mTOR pathway being crucial for the HB survival. Most tumors express p-GSK-3β, p-Akt and p-mTOR and some of them show a significant expression of cyclin D1, p21, and p27. 

It has been demonstrated that the inhibition of PI3K diminished the HB cell growth, this being accompanied by a reduced phosphorylation of both GSK-3β and Akt (Figure 1). The inhibition of PI3K increases the HB apoptosis and decreases the HB cellular proliferation, this being linked to increased p27 and reduced cyclin D1 levels [43]. In human HB HepT1, HepG2 and HuH6 cell lines, it has been reported that the Akt signaling pathway is a target of aprepitant [44]. After blockage of the NK-1R with aprepitant, both phosphorylation sites (S473, T308) of Akt and mTOR (S2448) become increasingly phosphorylated (that is, an activation of upstream Akt/mTOR signaling occurs), whereas downstream components of Akt/mTOR (4E-BP1/2, p70S6K) are downregulated [12,44]. In the nucleus of the HB cells and after administration of aprepitant, the presence of phosphor-Akt (S473) and phosphor-mTOR (S2448) has been reported [12]. In the nucleus, Akt can phosphorylate intracellular components promoting DNA repair/cell survival [45]. It has been suggested that the activation of Akt could be related to a cellular mechanism that avoids cell death and contributes to drug resistance, attenuating the antitumor efficacy of aprepitant [12,46,47]. This could explain why, in experimental animals, although aprepitant significantly reduced the volume of the HB tumor, a slight increase of the volume was observed after twenty days of treatment [6,12]. In summary, the data suggest that the PI3K/Akt/mTOR signaling pathway (Figure 1) plays a crucial role in the HB growth inhibition exerted by NK-1R antagonists.

### 3.2. Wnt Signaling Pathway

This pathway plays an important role in survival, differentiation and cell proliferation (Figure 1) [12]. In most HB, missense mutations/exon-3 deletions in gene coding for β-catenin (a downstream effector of the Wnt signaling pathway) have been reported and HB has been associated with an aberrant activation of the β-catenin pathway (Figure 1) [3,36]. The Wnt pathway is activated via inhibition of the β-catenin degradation; thus, the level of β-catenin is increased and then the protein is translocated into the nucleus promoting cell proliferation (Figure 1) [12]. Wnt3a is reported to activate the ERK pathway through Ras, Raf and mitogen-activated protein kinase kinase (MEK); that it is involved in cellular proliferation, and that it activates p38MAPK which is crucial for the accumulation of β-catenin (this accumulation promotes tumorigenesis) (Figure 1) [48]. Wnt signaling has been reported as corroborating an active canonical Wnt signaling and it is known that SP activates the Wnt signal transduction pathway and enhances the proliferation of stem cells (Figure 1) [49,50]. Wnt signaling is involved in the SP inhibition of apoptosis. SP exerts a protective effect, reduces the apoptotic rate, produces nuclear condensation and the activation of caspase-3 and caspase-9. The inhibition of Wnt signaling or NK-1R antagonists blocked this effect. SP promotes the mRNA and protein expression of Wnt signaling molecules such as β-catenin, GSK-3β, c-myc and cyclin D1 (Figure 1) [51]. 

In human HB HepT1, HepG2 and HuH6 cell lines treated with aprepitant, it has been demonstrated that the Wnt and Akt signaling pathways are the targets of NK-1R antagonists [44]. Aprepitant inhibited the canonical Wnt pathway (evidenced by the decrease of β-catenin and the downregulation of the Wnt target genes *AXIN2* and *LGR5*) (Figure 1); it decreased the phosphorylation of 4E-BP1/2 and p70S6K, and it decreased the growth of the HB cells [44]. Aprepitant is also involved in the downregulation of FOXM1 (a protein involved in the translocation of β-catenin into the nucleus) [12,52]. It seems that the inhibition of the Wnt pathway is strengthened by the FOXM1-β-catenin complex disruption, enhancing growth arrest and apoptotic mechanisms [44]. It is also important to note that aprepitant decreased stemness property/canonical Wnt signaling in Wnt-dependent cancer stem cells [12]. This is extremely important because it seems that cancer stem cells are involved in tumor relapse/resistance [53].

## 4. NK-1R Antagonists as Anti-HB Drugs: Mechanisms of Action and Therapeutic Properties

There are peptide and non-peptide NK-1R antagonists. Peptide NK-1R antagonists are degraded by peptidases, they are not brain-penetrant and some of them exert toxic effects, but non-peptide NK-1R antagonists (e.g., rolapitant (Varubi), aprepitant (L-754,030, MK-869, Emend), GR-205,171 (Vofopitant), CP-96,345, WIN-51,708, L-733,060, L-732,138) are not degraded by peptidases and they can cross the blood-brain barrier [7]. Thus, non-peptide NK-1R antagonists show more therapeutic advantages than peptide NK-1R antagonists which are toxic and unstable [54]. Only three NK-1R antagonists (non-peptide) have been approved for humans: Aprepitant, its pro-drug (fosaprepitant, MK-0517, L-758,298, Ivemend) and rolapitant (Varubi). SP binds to the extracellular loops of the receptor, but non-peptide NK-1R antagonists (lipid soluble) bind more deeply [55]. It is important to note that the therapeutic action of non-peptide NK-1R antagonists, after binding to the fl-NK-1R or the tr-NK-1R isoforms, is similar because such action is not associated with the intracellular C-terminus. Non-peptide NK-1R antagonists (e.g., aprepitant (morpholine derivative), L-733,060 (benzyletherpiperidine), L-732,138 (tryptophan-based)) are broad spectrum antitumor compounds that exert an antitumor action against many human cancer cells (e.g., HB, neuroblastoma, glioma, melanoma, retinoblastoma, acute lymphoblastic leukaemia B and T cells, gastric, colon, breast, larynx and pancreatic carcinoma) (Table 1) [4,6]. The antitumor action exerted by these antagonists is in a concentration-dependent manner: The higher the concentration, the greater the antitumor activity (Table 1) [4,6].

### 4.1. Antiproliferative Effect

Human HB cells overexpress the NK-1R (truncated form) and NK-1R antagonists (L-733,060, L-732,138, aprepitant) after binding to the NK-1R, exerting an antiproliferative action against the HB cells lines (HepT1, HuH6, HepG2) (Table 1) [6]. Thus, the IC_50_ of aprepitant for HB HepT1, HuH6 and HepG2 cells were 31.1 µM, 33.18 µM, and 38.61 µM respectively; 42 µM, 41 µM, and 110 µM (for L-732.138), and 15 µM, 14 µM, and 17 µM (for L-733,060) (Table 1) [6]. It seems that this effect is due to the blockade of the MAPK cascade (including p38MAPK and ERK1/2 signaling). It is known that SP, through the G-protein βγ subunit, which recruits components of the Ras-dependent cascade (e.g., SHC, GRB2, Src), leads to the activation of Raf-1 and MAPK 1, a specific activator of ERK1/2 (Figure 1) [56]. Thus, NK-1R antagonists could counteract this pathway. In a HuH6 xenograft mouse model (80 mg/kg/day aprepitant for 24 days), reduction of tumor growth (reduced tumor volume and weight) was reported along with a lowered tumor-specific alpha-fetoprotein (a marker of HB) serum level and a decreased number of Ki-67 positive cells (Table 2) [6]. In this model, NK-1R antagonists induced a dual effect: Decreased the volume of the tumor (tumor cells die by apoptosis, see below) and exerted an antiangiogenic effect (Table 2) [6].

### 4.2. Apoptotic Effect

NK-1R antagonists (L-733,060, L-732,138, aprepitant) promoted apoptosis in human HB HepT1, HuH6 and HepG2 cell lines (Table 1) [6]. Moreover, in HB, NK-1R antagonists increased the percentage of cells with subdiploid DNA features of apoptotic death; caused the cleavage of caspase-3 and the proteolysis of poly (ADP-ribose) polymerase (Table 1) [6]. This same NK-1R antagonist is known to promote apoptosis in many other tumor cells [4] and the blockade of this receptor by L-733,060 caused the cleavage of caspase-3 and the proteolysis of poly (ADP-ribose) polymerase, increased apoptosis and inhibited the basal kinase activity of Akt [57]. In the Akt phosphorylation mediated by the NK-1R, the full involvement of PI3K/the non-receptor tyrosine kinase Src was reported as well as the partial involvement of EGFR and the non-involvement of MAPK/ERK (Figure 1) [57]. It is known that the expression of the NK-1R is pivotal for the survival of cancer cells. By eliminating the expression of this receptor in cancer cells, due to apoptotic mechanisms, a decrease in the number of these cells has been reported and it is also known that in tumor cells the application of the si*RNA TAC1R* method decreased the levels of p-EGFR, p-Akt and p-ERK (Figure 1) [5,26,58]. 

### 4.3. Anti-Warburg Effect

In order to obtain energy, cancer cells show a high rate of glycolysis (200 times higher than those of their normal tissues of origin) followed by lactic acid fermentation (Warburg effect) [4,59]. In cancer cells, SP (via the NK-1R) increases the concentration of intracellular Ca^++^ (Figure 1), induces the breakdown of glycogen and then, due to the glucose obtained, these cells increase their metabolism; however, NK-1R antagonists block such a breakdown and cancer cells die from starvation [60]. The glycogen synthase is involved in glycogenesis, whereas glycogen synthase kinase-3 (GSK-3β) (Figure 1) inactivates the glycogen synthase and produces glucose. It has been reported that GSK-3β is linked to poor prognostic/cancer progression and that the inhibition of GSK-3β suppresses tumorigenesis by attenuating cell proliferation, while increasing apoptosis and restraining cell motility [61,62]. Inhibitors of GSK-3β reduce glucose output and increase the synthesis of glycogen from L-glucose, counteracting the Warbur effect and it is also known that SP activates GSK-3β and that NK-1R antagonists inhibit GSK-3β activity [57]. The previous data suggest that NK-1R antagonists could counteract the Warburg effect by inhibiting the GSK-3β activity in HB cells. This must be confirmed in future studies (Table 3).

### 4.4. Antiangiogenic Effect

Neoangiogenesis is crucial in advanced stages of the inflammatory process and in tumor growth development, this being associated with an increased innervation of the tissue and an overexpression of the NK-1R [4,28]. The increased level of SP found in chronic inflammatory conditions plays an important role in the development of new vessels [63]. Infiltrating immune cells express SP and NK-1R and the peptide exerts a pro-inflammatory action [12,64]. It is well known that the blood vessels located in the tumor/peritumoral region express both SP and NK-1R [28]. The SP/NK-1R system is involved in the proliferation of endothelial cells, followed by the formation of blood vessels (increasing vascularization) and by an increase in blood flow and hence neovascularization is crucial for the growth of the tumor mass [65]. However, NK-1R antagonists block such proliferation [4,66].

It has been reported that SP promotes the release of IL-1, IL-6, IL-10, IL-12 and TNFα from inflammatory cells; that SP controls both secretion and gene expression of the vascular endothelial growth factor (VEGF, a pro-angiogenic factor involved in many inflammatory diseases); that this secretion increased when SP and IL-33 were co-administered, and that NK-1R antagonists inhibited the previously mentioned effects exerted by SP [67]. Moreover, it is known that calcium-dependent protein kinase C isoforms, ERK, c-jun N-terminal kinases (JNK) and MAPKs are involved in the production of the VEGF regulated by SP [67]. SP is expressed in human HB tumors [6]. This suggests that via a paracrine action, the SP released from the HB cells could favor the vascularization of the HB tumor by stimulating, via the NK-1R expressed in endothelial cells, the proliferation of these cells. This paracrine action must be confirmed in future studies.

In a human pancreatic xenograft model, it has been demonstrated that a peptide NK-1R antagonist blocked the growth of the tumor by antiproliferative and antiangiogenic mechanisms [68]. This was also observed in a HB xenograft nude mouse model [6]. Thus, aprepitant blocked the HB growth by inhibiting both HB cell proliferation and angiogenesis (Table 2): A decrease of the vascularized area was observed, but not of the microvascular density [6]. The finding confirms that NK-1R antagonists counteract angiogenesis in HB.

### 4.5. Antimetastatic Effect

In advanced stages of HB, invasion and metastasis occur and unfortunately for 25% of the patients with HB metastasis, the overall survival remains poor [3]. Membrane blebbing plays an important role in cell movement/spreading and in tumor cell infiltration/invasion [69]. It is known that SP promotes the migration of tumor cells (SP favors the formation of membrane blebbing) for invasion/metastasis; that SP increased the expression of VEGF-C and matrix metalloproteinase favoring tumor metastasis; that NK-1R antagonists inhibit such an increase [70,71], and that these antagonists block the changes in the cell shape (including blebbing) induced by SP (in the latter mechanisms Rho-associated protein kinase (ROCK) was involved) (Figure 1) [72]. In tumor cells (but not in non-tumor cells), SP induced the phosphorylation of p21-activated kinase and augmented the phosphorylation of the myosin regulatory light chain kinase [73]. Moreover, the loss of the EGFR-ASAP-1 signaling has been reported to be an important characteristic of invasive/undifferentiated HB [74].

Since the HB cells express both SP and the NK-1R [6], it is possible that the peptide could trigger the migration of the HB cells for invasion/metastasis and that it could be blocked by NK-1R antagonists. This must be confirmed in the future (Table 3). 

### 4.6. Combination Therapy Using NK-1R Antagonists

In HB patients, the high-risk group is characterized by a marked chemoresistance and poor outcome [75]. Multidrug resistance often occurs after four cycles of chemotherapy [12]. In clinical practice, the main goal of the combination therapy is to increase the therapeutic effects (additive or synergistic) of a drug. In HB, the adjuvant chemotherapy regimens include cisplatin (induces ototoxicity/nephrotoxicity) [76], doxorubicin (DOX) (induces cardiotoxicity) [77] and microtubule destabilizing agent (MDAs, e.g., vinorelbine, vincristine) treatments. In human HB HepT1, HuH6 and HepG2 cell lines, it has been demonstrated that aprepitant and cytostatics showed a synergistic effect (Table 1) [6]. In HepT1 cells, a synergistic effect for DOX/aprepitant was found, but this effect was not observed in the case of cisplatin/aprepitant [6]. In HepG2, a synergistic effect was found for aprepitant/DOX or cisplatin [6]. Even the effects of aprepitant/DOX (low-dose) were significantly higher when compared to the treatment with single compounds. In HuH6, similar results were obtained [6]. These data suggest that in HB patients aprepitant, in combination therapy with the above-mentioned drugs, could exert a synergistic therapeutic effect. Moreover, it has been demonstrated that pre-treatment of non-tumor HEK-293 cells with a NK-1R antagonist (before exposure to cytostatics), protected the HEK-293 cells from cytostatic toxicity (Table 1) [33].

Treatment with DOX is known to elicit cardiotoxicity [77] and that the SP/NK-1R system mediates this DOX cardiotoxicity. In tumor cells, the treatment with aprepitant increased cell death by apoptosis, reactive oxygen species synthesis and DOX-induced reduction of cell viability, whereas in cardiomyocytes treatment with the same drug decreased apoptosis, reactive oxygen species synthesis and DOX-induced reduction of cell viability [78].

NK-1R antagonists and MDAs exert a synergic effect. MDAs trigger apoptotic signaling through JNK, and the modulation of the MAPK pathway can inhibit or enhance the apoptosis induced by MDAs. It seems that NK-1R antagonists sensitizes tumor cells to the MDA-mediated inhibition of cell viability by reducing the anti-apoptotic NK-1R signaling, thus enhancing the cell death induced by MDAs [79]. This combination is synergistic for the growth inhibition of cancer cells expressing the NK-1R, but not for non-cancer cells. Moreover, the combination therapy of NK-1R antagonists/cytostatics decreases the toxicity/side-effects of chemotherapy and it is less toxic than treatments with chemotherapy alone [79]. In summary, the strategy suggested might be clinically useful for those patients with cancer (e.g., HB) that must be treated with chemotherapy (Table 3).

Recently, a case report has been published on a patient with lung cancer that was treated with a combination therapy of aprepitant (1140 mg/day) and radiotherapy [80]. The patient was treated for 45 days and, six months after treatment, the tumor mass had disappeared [80]. Three years after the treatment, the patient died due to causes not related to cancer [80]. More studies must be performed to test combination therapy strategies. In addition, it is important to test the antitumor action of aprepitant when administered alone (it has been suggested 25 mg/kg/day, for a long period of time according to the response of the treatment) (Table 3) [81].

## 5. Safety Profile and Side-Effects of NK-1R Antagonists

Contrary to cytostatics, NK-1R antagonists at antiemetic doses do not produce serious side-effects, although headaches, hiccupping, vertigo and drowsiness have been reported after administration of these antagonists [82,83]. In patients with moderate to severe depression the safety of aprepitant was reported: A dose of 300 mg/day was tolerated very well while no statistically significant difference in the frequency of adverse events was observed as compared to the placebo [84]. The safety of aprepitant against human fibroblasts has also been demonstrated: The IC_50_ for tumor cells (HB HepT1, HuH6, and HepG2 cell lines were 28.5 μM, 31.1 μM and 33.18 μM, respectively) was lower than that for fibroblasts (57.5 μM) [6]. In addition, the safety of aprepitant against lymphocytes has been recently reported (IC_50_ 176.2 μM) [85].

As mentioned above, a patient with lung cancer was treated for 45 days with aprepitant (1140 mg/day) [80]. No side-effects were observed [80]. In this case report, the safety of aprepitant was demonstrated since no biochemical analytical alteration was observed and the patient showed very good general health, including weight gain [80]. This means that aprepitant is safe at the high dose necessary for the HB treatment (25 mg/kg/day) [81].

## 6. Conclusions

According to the data reported in previous sections, Table 3 shows the key-points in future HB research. Altogether, data show that NK-1R is a new therapeutic target for the treatment of HB because NK-1R antagonists (e.g., aprepitant) exert an antitumor effect (antiproliferative, apoptosis, tumor volume is decreased) against HB cells (they overexpress the NK-1R (truncated form) and the receptor must be used as a tumor marker). In addition, NK-1R antagonists decreased the angiogenic activity in HB. This means that NK-1R antagonists exert a dual antitumor action in HB: Antiproliferative and antiangiogenic, and that the SP/NK-1R system plays an important role in the tumor microenvironment. In HB patients, the NK-1R may serve as a therapeutic target, independent of the clinical stage. The tr-NK-1R isoform is expressed essentially among the different subsets of HB and it seems that this isoform is associated with cancer rather than the full-length isoform. It seems that HB cells have no possibility of becoming resistant to NK-1R antagonists since in HB cells the tr-NK-1R isoform is continuously up-regulated (the truncated isoform could be responsible for a constitutive growth stimulus) and, for this reason, the antitumor effect of the NK-1R antagonists is linked to the differential expression of the NK-1R (truncated and full isoforms) in cancer cells. In combination therapy, NK-1R antagonists (show no severe side-effects) and cytostatics produce an antitumor synergistic effect against the HB cells (this is important in order to decrease the concentration of cytostatics in clinical practice), decrease the toxicity of chemotherapeutics and increase the sensitivity of the HB cells to cytostatics. Since aprepitant targets two crucial pathways involved in tumorigenesis (PI3K/Akt/mTOR and Wnt), NK-1R antagonists could more easily block escape mechanisms in the HB cells and decrease HB relapse/resistance. In summary, NK-1R is a new marker and target for the treatment of HB and NK-1R antagonists exert an antitumor/antiangiogenic action against HB. 

## Figures and Tables

**Table 1:** The SP/NK-1R system in human HB cell lines (HepT1; HepG2; HuH6).

**NK-1R**	Express mRNA for the NK-1R
	Expression of the *TAC1R* gene is increased
	Express full and truncated isoforms of the NK-1R. The HB cells express essentially the truncated form. Expression of the full form is higher in non-tumor cells
**SP**	A universal mitogen (at nanomolar concentration) of tumor cells, including HB cells
**Non-peptide NK-1R antagonists**	Antiproliferative action in a concentration-dependent manner: The higher the concentration, the greater the antitumor activity
	Induce cell death by apoptosis, cleavage of caspase-3and proteolysis of poly (ADP-ribose) polymerase
	Aprepitant (IC_50_) for HepT1 (31.1 µM), HuH6 (33.18 µM), HepG2 (38.61 µM)
	L-732,138 (IC_50_) for HepT1 (42 µM), HuH6 (41 µM), HepG2 (110 µM)
	L-733,060 (IC_50_) for HepT1 (15 µM), HuH6 (14 µM), HepG2 (17 µM)
	Co-administration of aprepitant and cytostatics exerts a synergistic antitumor effect
	Pretreatment of non-tumor cells (human embryonic kidney (HEK)-293) with aprepitant, protected these cells from cytostatic toxicity

**Table 2:** The SP/NK-1R system in the human HB sample and tumor.

Items	HB Sample	HB Tumor
**SP/NK-1R system**	Expression of both SP and NK-1R	Truncated-*TACR1* isoform is expressed ubiquitously among the different subsets of children with HB
**Non-peptide NK-1R antagonists**		In experimental animals: Dual effect of aprepitant (80 mg/kg/day for 24 days). The antagonist decreased the tumor volume (tumor cells die by apoptosis) and the angiogenic activity (decrease of the vascularized area, but not the microvascular density). Aprepitant lowered the tumor-specific alpha-fetoprotein serum level and decreased the number of Ki-67 positive cells

**Table 3:** Key points in future HB research.

**NK-1R**	NK-1R: A new HB tumor marker
	NK-1R: Therapeutic target in HB patients, independent of the clinical stage/tumor biology
	NK-1R: Involved in the viability of HB cells?
	The tr-NK-1R, but not the fl-NK-1R, is associated with cancer?
	The response of the NK-1R antagonists depends on the differential expression of the receptor (truncated/full)?
	tr-NK-1R: Responsible for a constitutive growth stimulus in the HB cells?
	Overexpression of the tr-NK-1R: Correlates with poorer prognosis and advance HB stages?
	tr-NK-1R: Not internalized in the HB cells? Involved in the malignant transformation of the HB cells?
**SP**	Level of SP in the serum of HB patients
	SP activates GSK-3β in the HB cells (Warburg effect)?
	SP is released from the HB cells to promote neovascularization?
	SP triggers the migration of HB cells?
